# Guillain–Barré Syndrome Associated with COVID-19: Two Cases from a Public Hospital in Damascus, Syria

**DOI:** 10.1155/2022/9722736

**Published:** 2022-09-02

**Authors:** Tagrid Ahmad, Somar Hamdan, Imad Saadeh

**Affiliations:** ^1^Department of Neurology, Tishreen Military Hospital, Damascus, Syria; ^2^Damascus Medical Association, Damascus, Syria

## Abstract

**Objective:**

To report two cases of COVID-19 complicated with Guillain–Barré syndrome (GBS) from a public hospital in Damascus, Syria.

**Results:**

Two unvaccinated patients, 49-year-old and 34-year-old men, presented with a history of paresthesia followed by ascending symmetric weakness with absent tendon reflexes. They were diagnosed with coronavirus disease 2019 (COVID-19) using chest CT and RT-PCR. Clinical status and lumbar puncture (LP) findings were consistent with GBS. They were treated with plasma exchange (PE). However, the first patient developed hallucinations and later deteriorated. He passed away after the second session of PE, while the second patient had four sessions of plasma exchange and was later discharged for home rehabilitation.

**Conclusion:**

It is important to consider GBS in COVID-19 patients who present with acute ascending weakness or cranial nerves involvement. More studies are needed to evaluate correlation between COVID-19, GBS, and development of psychiatric disturbances besides investigating the discrepancy between lung parameters and respiratory failure.

## 1. Introduction

The coronavirus disease 2019 (COVID-19) pandemic threat has led to more than 245,373,039 confirmed cases and 4,979,421 deaths globally as of 30 October 2021 [1]. Neurological manifestations have become more frequent, affecting central as well as peripheral nervous systems. Hypogeusia, hyposmia, and headache are among the cardinal presentations [2]. Furthermore, more serious complications have been described during the course of illness, such as cerebrovascular disease (CVS), neuromuscular diseases, encephalopathy, and meningitis/encephalitis [2, 3]. Guillain–Barré syndrome (GBS) was reported in some cases, first in Italy by Toscano et al. as they reported five cases of COVID-19 with GBS [4]. Here, we report two cases of COVID-19 complicated by GBS from a public hospital in Damascus, Syria.

## 2. Case I

A 49-year-old male patient presented to the emergency department of Tishreen Military Hospital with a one-day history of ascending paresthesia followed by ascending symmetric weakness. Ten days prior to presentation, he had upper respiratory infection symptoms with a positive RT-PCR nasopharyngeal swab for severe acute respiratory syndrome coronavirus 2 (*SARS-CoV-2*) infection. He was unvaccinated. In the emergency department, on neurological examination, there was no cranial nerve involvement. However, motor examination revealed quadriparesis: Medical Research Council Grade (MRC) is 4/5 in upper limbs, while it is 2/5 in the lower limbs. Tendon reflexes were all absent. Hughes score was 4/6. Computed tomography (CT) of chest demonstrated bilateral glass ground opacities. Antibodies test of *SARS-CoV-2* was positive for both Immunoglobulin-M (IgM) and Immunoglobulin-G (IgG). Laboratory findings showed leukocytosis (WBC: 14980 C/µL) with mild lymphopenia (1100 C/µL), slightly elevated erythrocyte sedimentation rate (ESR: 24 mm/hr1), C-reactive protein (CRP: 7 mg/dL; reference range: up to 5 mg/dl), ferritin (398.6 ng/ml; reference range: 12–300 ng/ml), and lactate dehydrogenase (LDH: 536 U/L; reference range: 140–280 U/L). Lumbar puncture (LP) revealed marked elevation of CSF protein level (1.82 g/L) and no cell. Nerve conduction studies (NCS) were not performed. Clinical and laboratory findings were consistent with acute demyelinating polyradiculoneuropathy/GBS (Brighton criteria 2). Plasma exchange (PE) (50 ml/Kg) was started with four sessions planned, one every other day.

Two days later, after the second session of PE, paresis progressed to become MRC 1/5 in all extremities. He developed dysphagia, bilateral peripheral facial nerve palsy, and dyspnea. One day later, he developed confusion and episodes of visual and auditory hallucinations. Then, after the third session, high-grade fever was observed, and LP was repeated, which showed classic albuminocytologic dissociation with elevation in protein levels (2 g/L) with no cell. Arterial blood gases and laboratory tests were within normal limits. Few hours after PE, gas exchange and blood pressure worsened dramatically with sudden desaturation that required intubation. He was treated for multi-organ failure with no improvement. He died the next day.

## 3. Case II

A 34-year-old male patient presented to the emergency department with a 5-day history of ascending symmetric numbness and tingling, followed by severe distal weakness of the lower limbs in addition to severe dysesthesia. Three weeks earlier, he had upper respiratory infection symptoms. He had not been vaccinated for SARS-CoV-2.

On admission, the patient was febrile (38.5 C). On neurological examination, cranial nerves were spared. Examination of lower limbs showed distal paralysis (MRC 0/5) with proximal paresis (MRC 4/5), knee jerk reflexes were decreased, and the ankle jerk reflexes were absent, while upper limbs were normal. Hughes score was 3/6.

Chest CT scan demonstrated bilateral glass ground opacities. *SARS-CoV-2* RT-PCR nasopharyngeal swab was positive. Lumbar puncture revealed high cerebrospinal fluid (CSF) protein (0.51 g/L) and normal cell count (5/µL). Nerve conduction studies (NCS) of four extremities were consistent with acute motor-sensory axonal neuropathy (AMSAN) subtype, predominantly in lower limbs (Table 1).

Nerve conduction studies show reduced sensory action potientals in lower limbs with absent motor action potentials. In upper extremities, there is prologation in distal motor latencies for both median nerves. Findings are consistent with acute diffuse motor-sensory neuroradiculopathy.

These clinical findings with the NCS and CSF findings were consistent with GBS (Brighton criteria 1). Two days later when the fever resolved, he underwent 4 sessions of plasma exchange (50 ml/kg), one every other day. After the fourth session, there was no evident clinical change.

Because he was stable, he was discharged four days after the end of plasma exchange course and continued rehabilitation at home.

## 4. Discussion

Severe acute respiratory syndrome coronavirus 2 (*SARS-CoV-2*) is considered to be related to severe acute respiratory syndrome (SARS) and Middle East respiratory syndrome (MERS) viruses as it has similar nucleotide sequences with other SARS-like coronaviruses, reaching 89.1% [3, 5]. They share similar viral structure as well as receptor-binding domain [5]. In general term, they are neurotropic viruses with studies suggesting two routes of invasion into the nervous system [5]. First is the retrograde axonal route and second is hematogenous [3, 5]. They act through specific receptors: angiotensin converting enzyme-2 (ACEII), which are present in respiratory epithelium as well as glial cells in brain and spinal nerves, thus becoming a target to these viruses [3, 5]. Reviewing literature, there are some studies suggesting that beta coronaviruses MERS-CoV may cause GBS. It was suggested that there is a prominent post-infection immune-mediated mechanism, besides the release of cytokines, which are massively released contributing to the amplification of dysimmune response in GBS [6]. On the other hand, other studies regarded this relation unlikely and no homology was found between SARS-CoV-2 surface epitopes and peripheral nerve tissue [7]. In some studies, virus was not observed in CSF analysis; besides, no solid evidence is available about direct invasion of peripheral nerves [8]. So, the exact relation and mechanism are not yet fully determined. Later, other cases of GBS were reported post some types of SARS-CoV-2 vaccines [9]. We presented two cases of GBS that may relate to COVID-19. Comparing information of our patients (age, time from onset of systemic illness to the manifestation of neurological symptoms, CSF findings plus electrophysiological findings) were all similar to previously reported cases worldwide. Generally, age of patients ranged between 23 and 77 years old, while a review of 73 cases reported mean age of 55 [6, 10]. Time from onset of systemic disease to clinical GBS ranged between 3 and 28 days [10]. Most cases of COVID-GBS were parainfectious [10]. Few cases were preceded by COVID-19 by only few days [10]. Clinical features of the COVID-GBS patients did not differ from those reported with other causes as sensorimotor syndrome was the most dominant compared to other variants as Miller Fischer [6]. In another study from Italy including 34 patients with COVID-19, AIDP was significantly more frequent in COVID-19-positive patients, while AMAN had higher rate in COVID-19-negative patients [8]. Occasionally, there were some cases reporting involvement of cranial nerves, ataxia, and gait abnormalities at presentation or during the course of the disease [6, 11]. There was rarity in autonomic disturbances [6]. Besides, there was worse MRC score or outcome (admission to ICU and arterial hypotension) in GBS following COVID-19 [8]. The presence of respiratory involvement was notable; a previous review of 39 patients reported respiratory failure in about 40% of patients compared to 20–30% in previous GBS patient cohorts [10]. Tassorelli et al. published an observation that may explain the discrepancy between lung parameters and chest imaging; they suggested a mechanism of direct invasion of *SARS*-*CoV*-*2* virus to cardiopulmonary centers in brainstem, supported by autopsy results of many patients [11]. Nonetheless, further studies are required [11]. Other causes may include GBS affecting respiratory muscles. These two possibilities may explain rapid deterioration of the first case we presented. Considering CSF analysis, reviews showed typical findings of albuminocytologic dissociation as seen in non-COVID-GBS patients [10]. Electro-neurophysiological studies showed predominance of demyelinating polyradiculoneuropathy, followed by axonal type; however, only few cases were compatible with mixed pattern [6]. A distinguishable point reported here is the development of hallucination in the first case. Unfortunately, rapid deterioration prevented from determining possible causality. Searching literature, several theories have been postulated. On the one hand, this may reflect psychological complications or presentations of COVID-19 [12]. Hallucination was found in 11% of patients, including visual as well as auditory types, and was related to delirium or more probably neuroinflammatory process [12]. On the other hand, it was not unusual to note the onset of psychiatric symptoms during acute phase of GBS, although it might be interpreted as intensive care unit (ICU) delirium [13]. These symptoms included visual hallucinations, paranoid delusions, disorientation, and psychosis [13]. Risk factors were autonomic dysfunction, assisted ventilation, and high CSF protein [13]. One of the earliest cases published on this topic suggested that sensory and motor denervation in GBS might cause confusion in thoughts that are generated internally and externally, similar to ones studied in alteration of sleep sensations [14]. This results in hallucinations and dream-like thoughts [14].

## 5. Conclusion

It is evident that *SARS*-*CoV*-*2* may have variable neurological manifestations. The present cases emphasize the importance of considering GBS in COVID-19 patients who present with acute ascending weakness or cranial nerve involvement. Nonetheless, more studies are needed to evaluate correlation between COVID-19, GBS, and development of psychiatric disturbances, as well as to investigate the discrepancy between lung parameters and respiratory failure.

## Figures and Tables

**Table 1:** Motor and sensory nerve conduction studies.

Nerve	Latency 1 (ms)	Latency 2 (ms)	Amplitude (mV)	Conduction velocity (m/s)	F-wave latency
Motor nerve conduction studies					
Medianus motor right					
Mid-palm	3.7	15.5	6.0	50	30.8
Wrist	9.1	12.1	5.2		
Medianus motor left					
Mid-palm	4.6	8.7	1.2	67	31.0
Wrist	8.2	11.3	1.0		
Ulnaris motor right					
Wrist	3.0	11.2	7.7	-	29.8
Below elbow-wrist	7.8	10.3	6.3	50	
Above elbow	9.7	10.8	6.2	53	
Ulnaris motor left					
Wrist	2.8	19.6	7.5		29.5
Below elbow-wrist	7.3	18.1	5.7	53	
Above elbow		17.6	6.0	50	
Peroneus motor left					
Ankle	—	—	—	—	
Fibula (head)	—	—	—	—	
Pop fossa	—	—	—	—	
Peroneus motor right					
Ankle	—		—	—	
Fibula (head)	—		—	—	
Pop fossa	—		—	—	
Tibialis motor left					
Ankle	—	—	—	—	
Pop fossa	—	—	—	—	
Tibialis motor right					
Ankle	—	—	—	—	
Pop fossa	—	—	—	—	
Sensory nerve conduction studies					
Medianus sensory Right					
Digit II-wrist	2.4	4.4	17	53	
Ulnaris sensory Right					
Digit V-wrist	2.2	3.7	11	50	
Radialis sensory right					
Digit V-wrist	—	—	—	—	
Suralis sensory left					
Ankle	2.7	4.5	8	56	
Suralis sensory right					
Lat. malleolus	2.8		10	54	
Peroneus superficialis sensory right					
Ankle	3.1	6.1	2	52	
Peroneus superfic sensory left					
Ankle	3.1	6.0	1	39	

## Data Availability

The data used to support the findings of this study are available from the corresponding author upon request.
